# Supplementary material for: Mechanism of U6 snRNA oligouridylation by human TUT1
Source: Nat Commun. 2023 Aug 10;14:4686. doi: 10.1038/s41467-023-40420-9 (PMC10415362; doi:10.1038/s41467-023-40420-9)
Supplement: Supplementary file 1 — Supplementary Information [file 41467_2023_40420_MOESM1_ESM.pdf]

Supplementary Information

Mechanism of U6 snRNA oligouridylation by human TUT1

Seisuke Yamashita[1] and Kozo Tomita[1*]

1. Department of Computational Biology and Medical Sciences, Graduate School of Frontier Sciences, The University of Tokyo, Kashiwa, Chiba, 277-8562, Japan

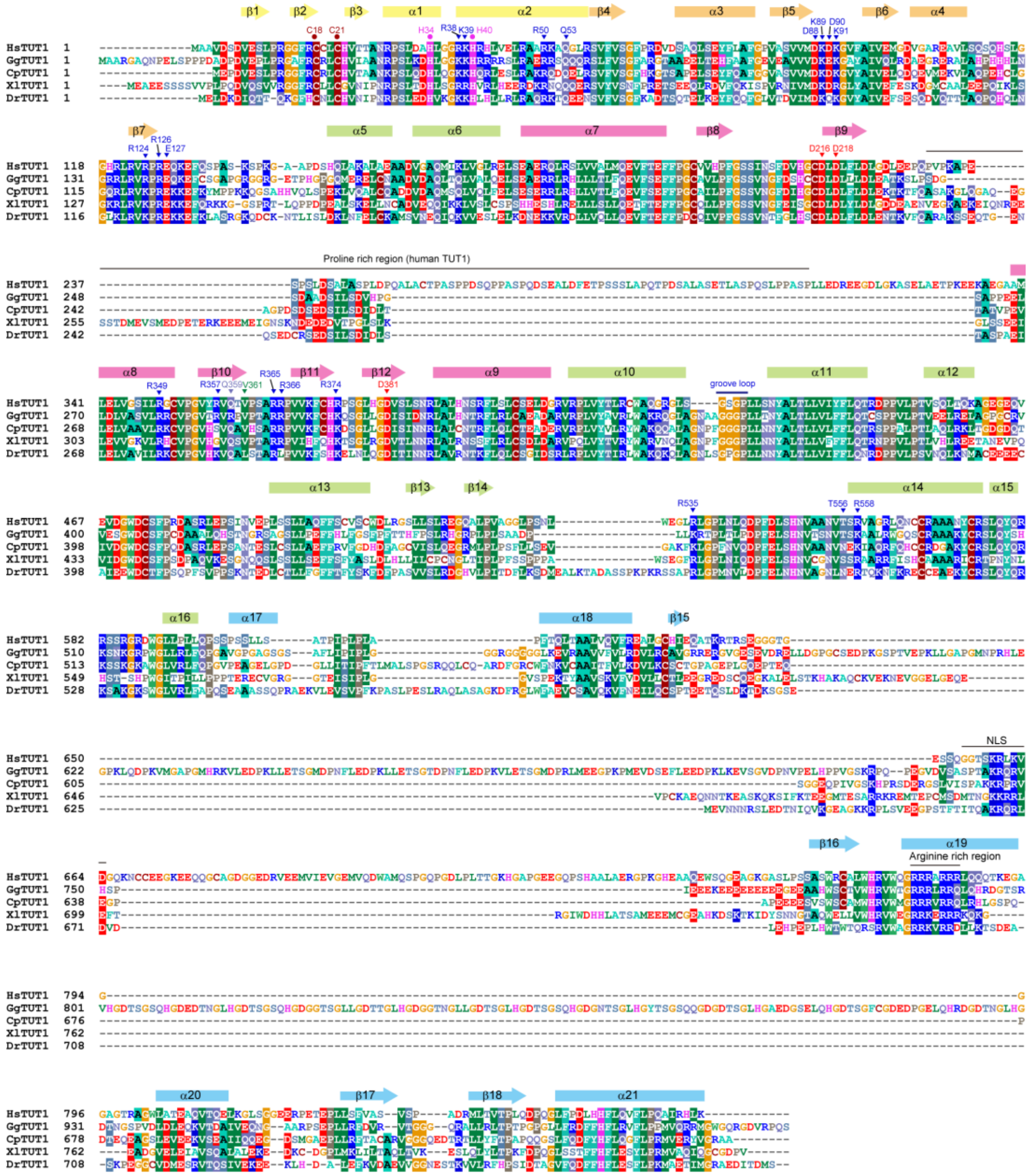

Supplementary Fig. 1: Alignments of the amino acid sequences of vertebrate TUT1s. The amino acid sequence of human TUT1 (HsTUT1, *Homo sapiens*; NP_073741.3) was aligned with those of other vertebrate TUT1 homologs. GgTUT1, *Gallus gallus* (XP_015128520.1); CpTUT1, *Chrysemys picta* (XP_008172327.1); XlTUT1, *Xenopus laevis* (XP_002941502.2) and DrTUT1, *Danio rerio* (NP_001025359.1). Secondary structures (α-helices and β-sheets) of human TUT1 domains are depicted above the sequences: ZF (yellow), RRM (orange), palm (magenta), fingers (green) and KA-1 (cyan). The proline-rich region (PRR), nuclear localization signal (NLS), and arginine-rich region in human TUT1 are underlined.

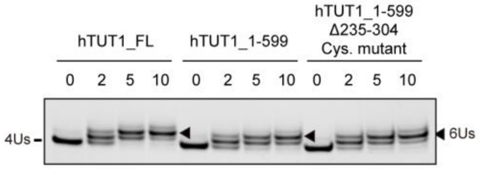

Supplementary Fig.2 Oligouridylation of U6 snRNA. Oligouridylation of U6 snRNA with 4Us by hTUT1_FL and its variants. hTUT1_1-599Δ235-304 Cys-mutant was used for crystallization in this study. U6 snRNA (200 nM) was incubated with 20 nM hTUT1_FL, hTUT1_1-599, and hTUT1_1-599_Δ235-304_Cys mutant (C19S/C372A/C415A/C501A/C504S/C399A/C574A) in the presence of 1 mM UTP. The arrowheads are reaction products with six uridines (6 Us) at their 3´-ends. The experiments were performed twice, and the representative gel image is shown. Source data are provided as a Source Data file.

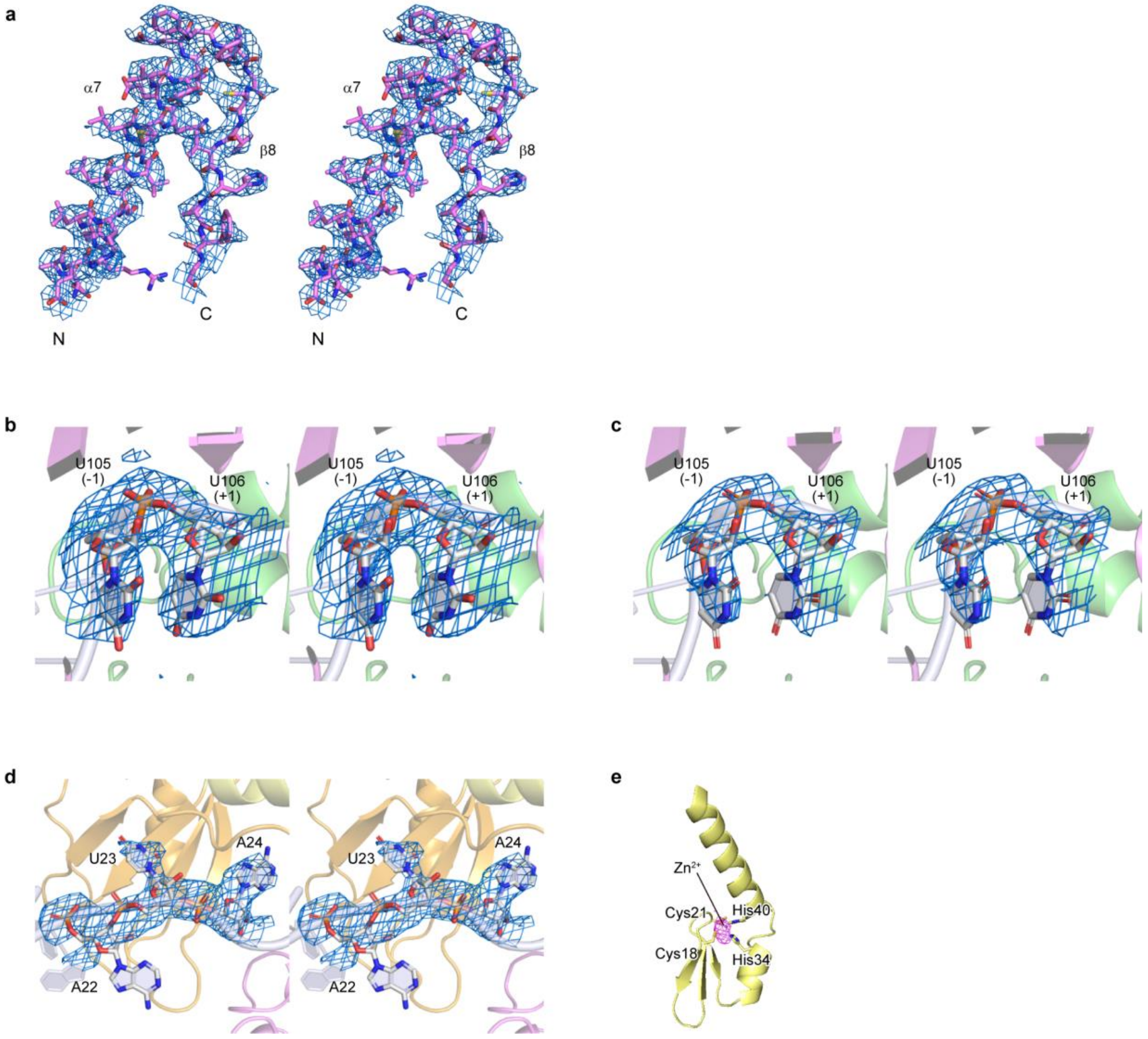

Supplementary Fig. 3: Electron density maps. (**a**) 2*F*o-*F*c electron density map contoured at 1.0 σ around the residues corresponding to α7 and β8 (residues Glu174 - Gly204). (**b**) 2*F*o-*F*c electron density map contoured at 1.0 σ around the 3'-terminus of U6_mini (nucleotides U105 and U106). (**c**) *F*o-*F*c omit map contoured at 2.0 σ around the 3'-terminus of U6_mini (nucleotides U105 and U106). (**d**) *F*o-*F*c omit map contoured at 2.0 σ around the AUA motif (A22U23A24). (**e**) The anomalous difference map (magenta) contoured at 2.5 σ around the zinc atom.

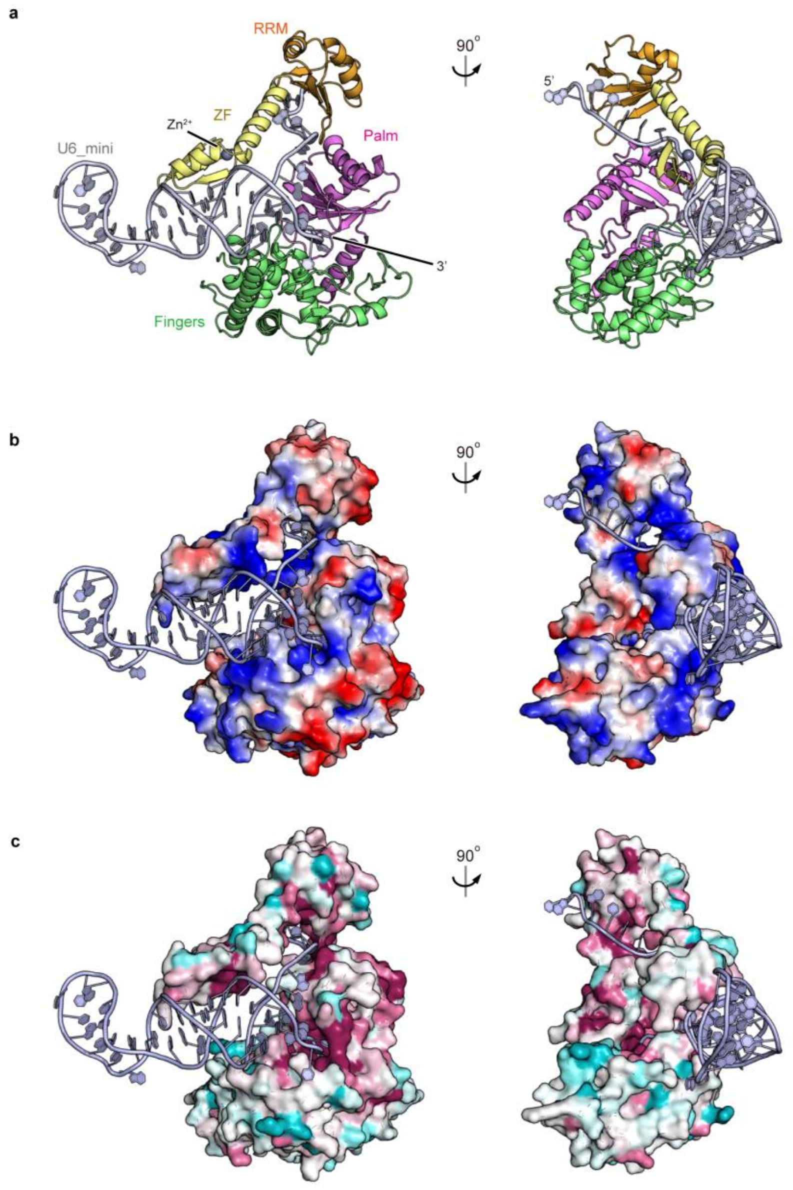

Supplementary Fig. 4: Overall structure of the hTUT1_ΔC- U6_mini complex. (**a**) Cartoon representation of the structure. ZF, RRM, palm, and fingers are colored yellow, orange, magenta, and green, respectively. U6_mini is colored gray. (**b**) The electrostatic surface potential of hTUT1_ΔC. The positively and negatively charged regions are colored blue and red, respectively. (**c**) Conservation analysis of hTUT1. Conserved and non-conserved residues are colored purple and cyan, respectively.

.

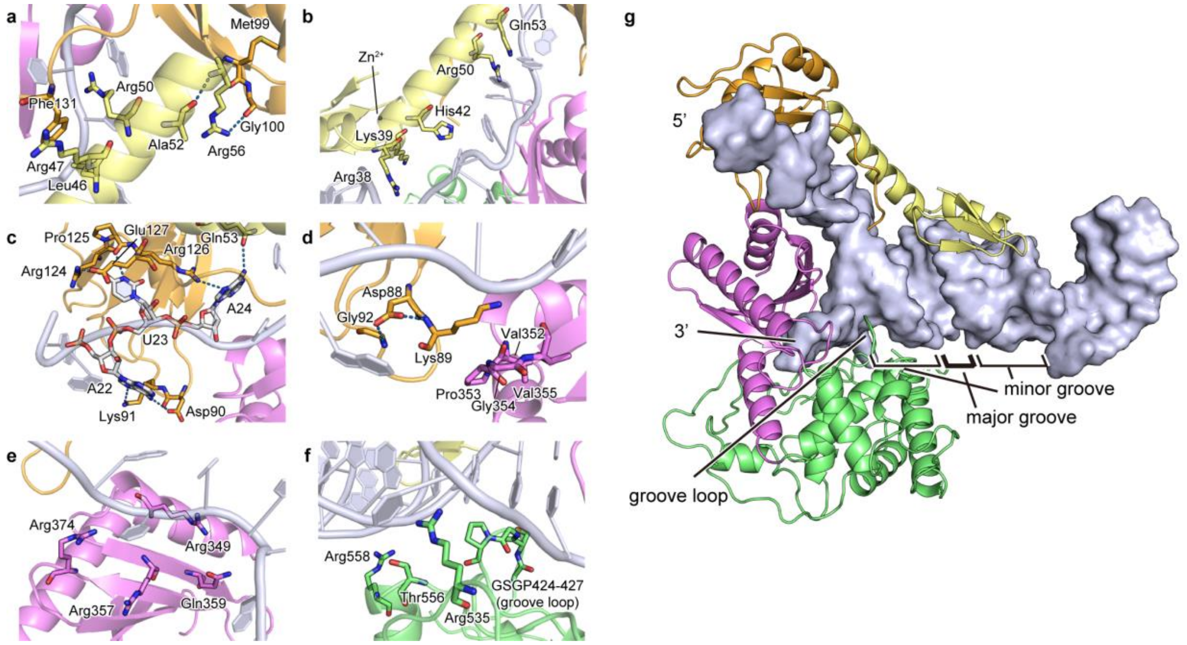

Supplementary Fig. 5: Interactions between hTUT1 and U6 snRNA. (**a**) - (**f**) Detailed views of the interactions between hTUT1_ΔC and U6_mini. (**g**) Recognition of U6_mini by hTUT1_ΔC. U6_mini is shown in a surface model (gray). The ZF (yellow) and the groove loop (amino acid residues 423 - 427) in the fingers (green) interact with the major and minor grooves of the telestem of U6_mini, respectively.

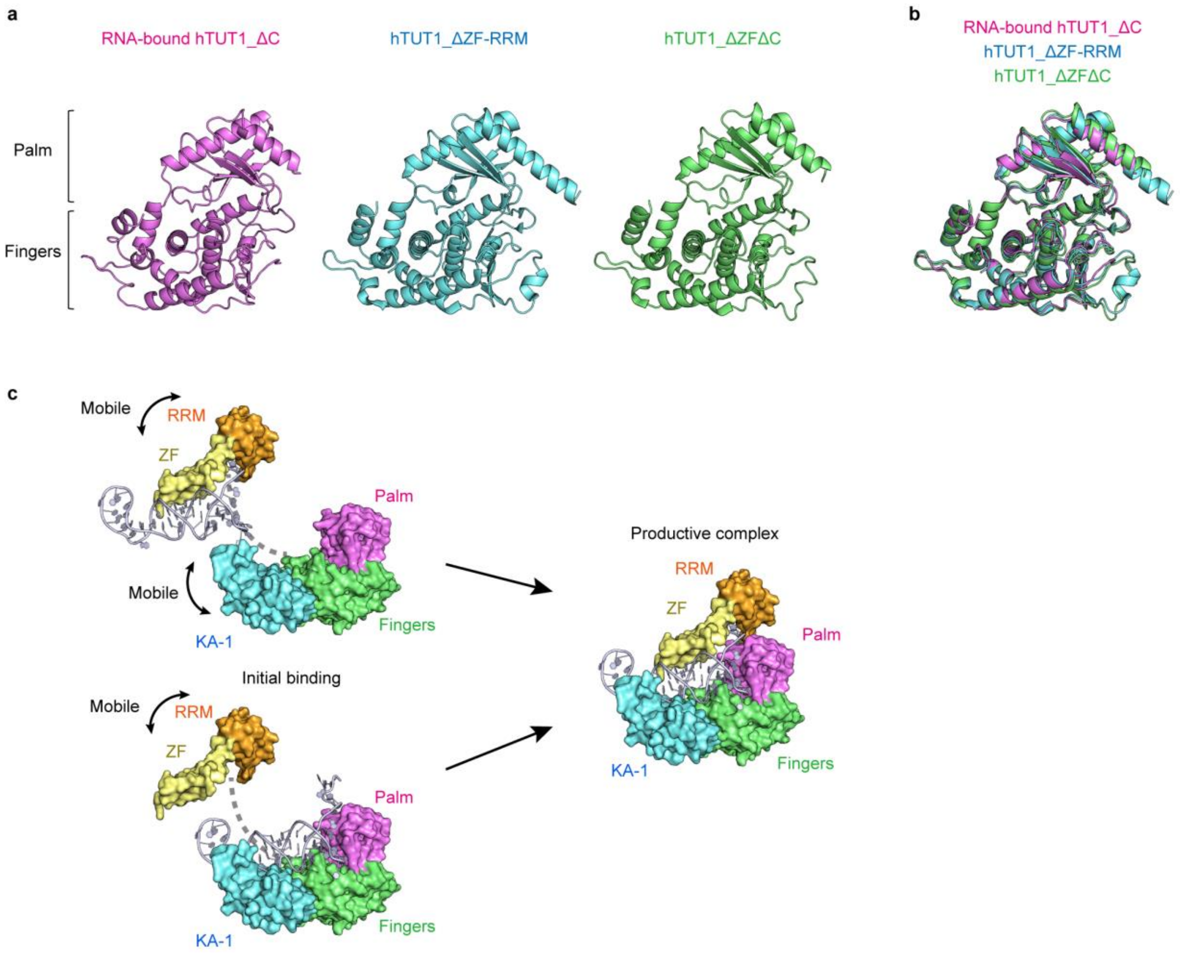

Supplementary Fig. 6: Comparison of hTUT1 core structures. (**a**) Structures of hTUT1 catalytic core domains from hTUT1_ΔC in complex with U6_mini (left, magenta), hTUT1_ΔZF-RRM (middle, cyan), and hTUT1_ΔZFΔC (right, green). (**b**) Superimposition of the catalytic core structures shown in (a). (**c**) Domain rearrangement of hTUT1 upon interactions with U6 snRNA. ZF, RRM, palm, fingers and KA-1 are colored yellow, orange, magenta, green, and cyan, respectively.

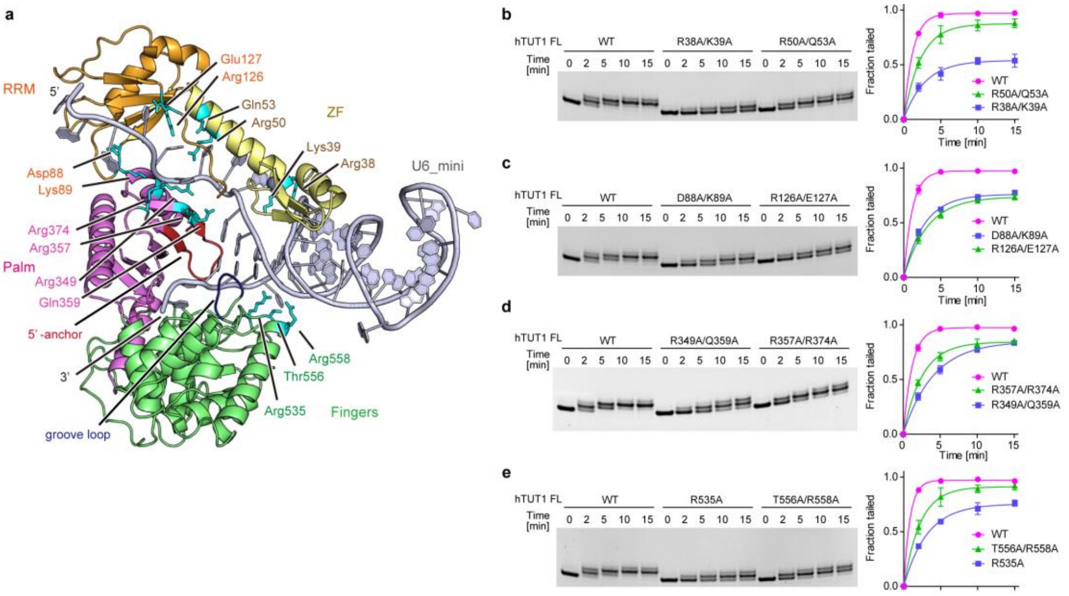

Supplementary Fig 7: *In vitro* uridylation of U6 snRNA by hTUT variants. (**a**) Overall structure of the hTUT1_ΔC-U6_mini complex. ZF, RRM, palm, and fingers are colored yellow, orange, magenta, and green, respectively. RNA-binding residues analyzed by the *in vitro* uridylation assay are shown as cyan sticks. (**b**) - (**e**) *In vitro* uridylation of U6 snRNA with four 3'-Us by hTUT1_FL and its variants. The U6 snRNA (200 nM) was incubated with 20 nM hTUT1_FL and its variants in the presence of 1 mM UTP at 37 °C. The reaction products were separated on a sequencing gel under denaturing conditions and stained with ethidium bromide (left). The tailed fractions were quantified (right). The experiments were performed three times, and the data are presented as mean values ± SD. Source data are provided as a Source Data file.

a

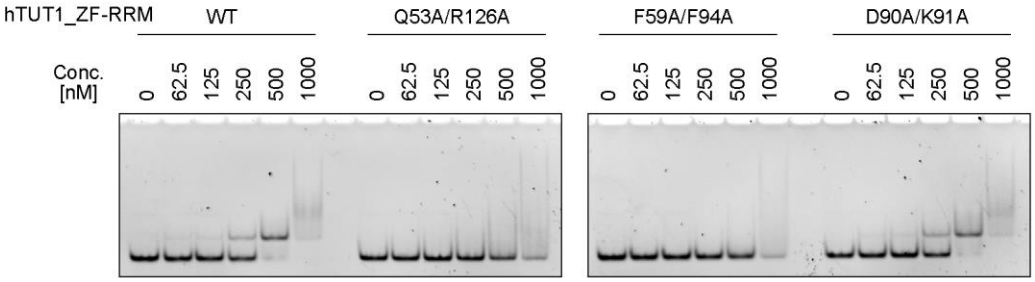

b

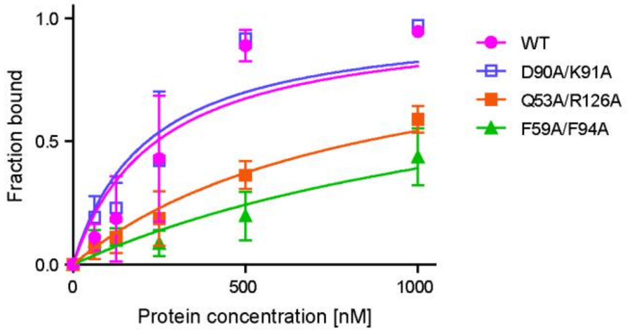

Supplementary Fig. 8: Gel shifts of U6 snRNA by hTUT1_ZF-RRM and its variants. (**a**) Gel shifts of U6 snRNA by hTUT1_ZF-RRM and its variants. U6 snRNA (50 nM) was incubated with various amounts of hTUT1_ZF-RRM and its variants (0 - 1,000 nM), and bound and unbound RNAs were separated by polyacrylamide gel electrophoresis under native condition. (**b**) The fractions of shifted RNA in the gels (a) were quantified. The experiments were performed three times, and the data are presented as mean values ± SD. Source data are provided as a Source Data file.

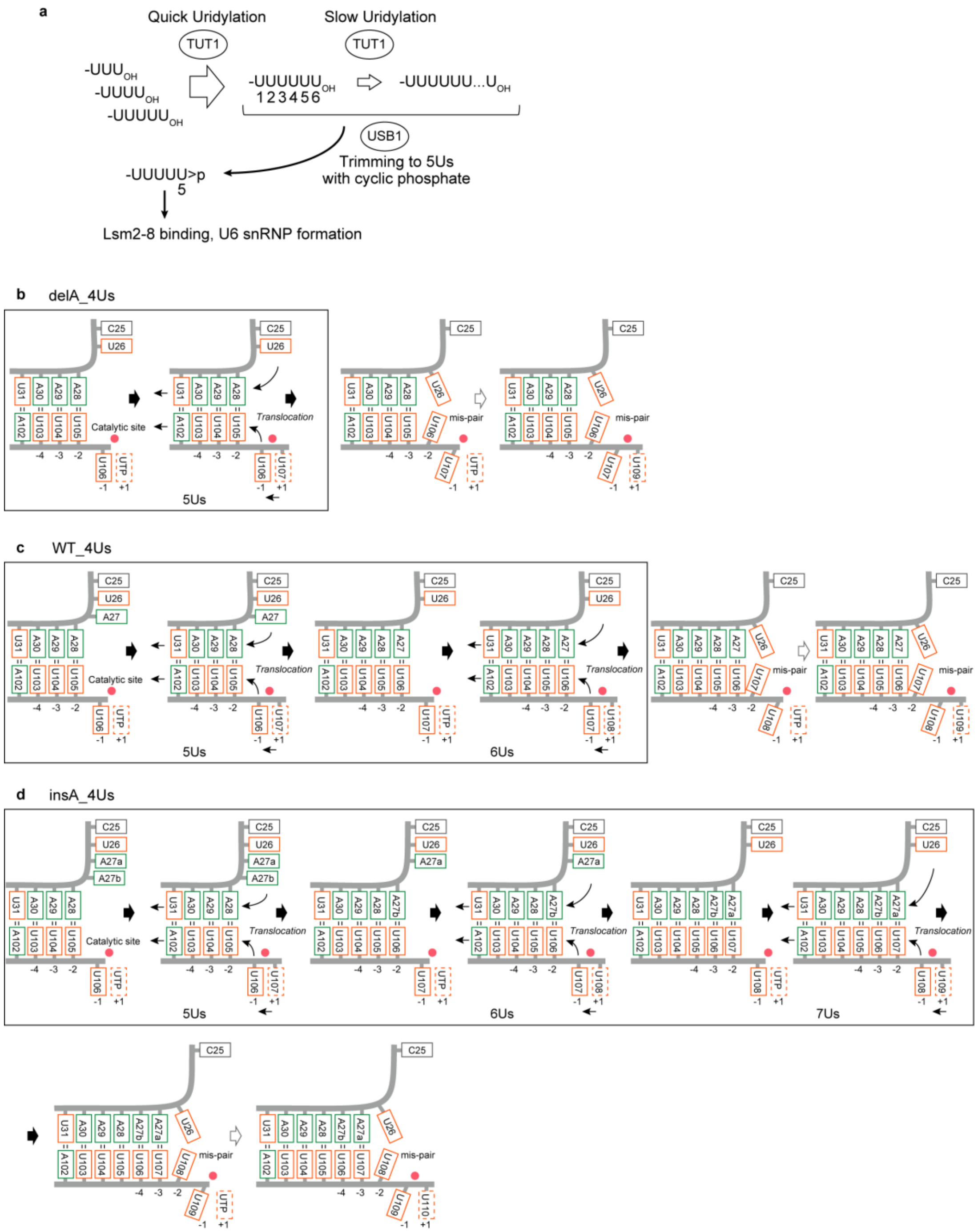

Supplementary Fig. 9: Model of U6 snRNA oligouridylation by hTUT1. (**a**) Schematic representation of the maturation process of U6 snRNA. (**b**) Oligouridylation of mutant delA_4Us by hTUT1. (**c**) Oligouridylation of wild type (WT_4Us) by hTUT1. (**d**) Oligouridylation of mutant insA_4Us by hTUT1.

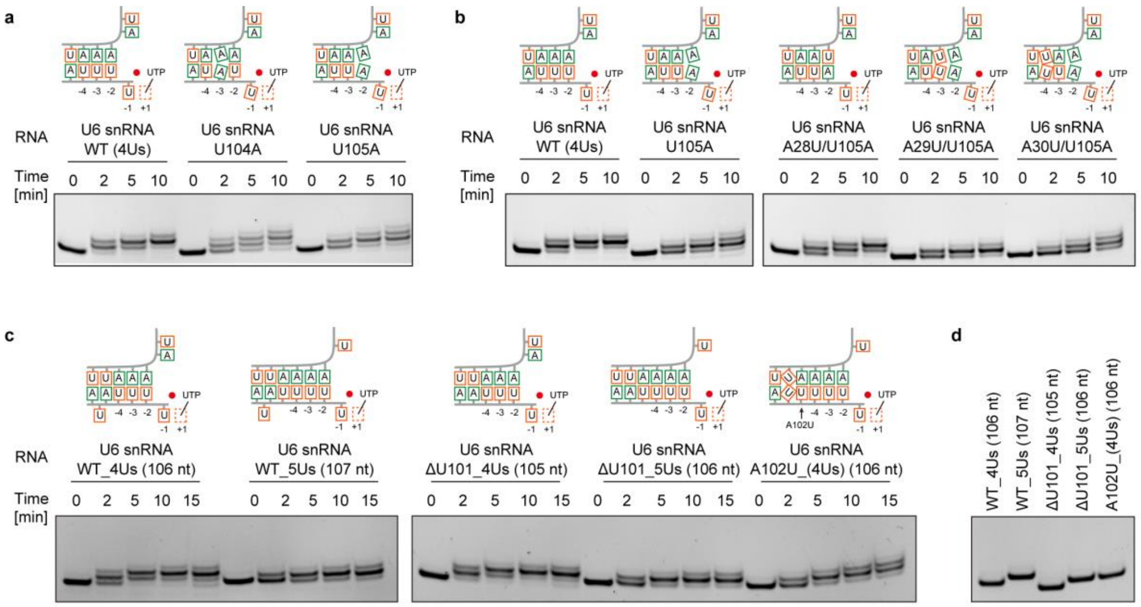

Supplementary Fig. 10: Oligouridylation of U6 snRNAs with variations in the telestem. (**a**) - (**c**) Oligouridylation of U6 snRNA variants. Schematic representations of the telestem regions of U6 snRNA variants are shown. U6 snRNA variants (200 nM or 100 nM) were incubated with 20 nM (or 10 nM) hTUT1_FL in the presence of 1 mM UTP at 37 °C. The reaction products were separated on a sequencing gel under denaturing conditions and stained with ethidium bromide. The experiments were performed three times, and the representative gel images are shown. (**d**) RNAs used in (c). Source data are provided as a Source Data file.

Supplementary Table 1. Data collection and refinement statistics

	hTUT1_ΔC- U6_mini
**Data collection**	
Space group	$P6_522$
Cell dimensions	
a, *b*, *c* (Å)	78.72, 78.72, 370.27
Wavelength (Å)	1.2700
Resolution (Å)*	50-3.7 (3.83-3.70)
R_{sym}*	0.534 (4.201)
$\langle I / \sigma I \rangle$ *	11.9 (2.1)
$CC_{1/2}$*	0.997 (0.819)
Completeness (%)*	99.5 (95.0)
Redundancy*	72.2 (76.9)
**Refinement**	
Resolution (Å)	20-3.7
No. reflections	7918
R_{work} / R_{free} (%)	30.17/33.86
No. atoms	
Protein	3529
RNA	1127
Zn	1
B-factors (Å^2)	
Protein	165.47
RNA	345.15
Zn	169.42
R.m.s. deviations	
Bond lengths (Å)	0.002
Bond angles (°)	0.50
Ramachandran plot	
Favoured	429 (95%)
Allowed	22 (5%)
Outliers	0
Rotamer outlier	0
Clash score	9

*Values in parentheses are for the highest-resolution shell.

Supplementary Table 2: Nucleotide sequence of the synthetic hTUT1 gene and its variants

human TUT1 gene	ATGGCGGCGGTTGATAGCGATGTTGAAAGCCTGCCGCGCGGTGGCTTTCGCTGCTGCCTGTGCCATGTGACC<br>ACCGCGAATCGCCCGAGCCTGGATGCACATCTGGGTGGCCGCAAACATCGTCATCTGGTGGAACTGCGTGCG<br>GCGCGTAAAGCGCAGGGCCTGCGTAGCGTGTTTGTGAGCGGCTTTCCGCGCGATGTGGATAGCGCGCAGCTG<br>AGCGAATATTTTCTGGCGTTTGGCCCGGTGGCGAGCGTGGTGATGGATAAAGATAAAGGCGTGTTTGCGATT<br>GTGGAAATGGGCGATGTGGGCGCGCGTGAAGCGGTTCTGAGCCAGAGCCAGCATAGCCTGGGCGGTCATCG<br>TCTGCGTGTGCGTCCGCGCGAACAAAAAGAATTTCAGAGCCCGGCAAGCAAAAGCCCGAAAGGCGCAGCG<br>CCGGATAGTCATCAGCTGGCGAAAGCGCTGGCGGAAGCAGCGGATGTGGGTGCGCAGATGATTAAACTGGT<br>GGGCCTGCGTGAACTGAGCGAAGCGGAACGCCAACTGCGCAGCCTGGTGGTGGCGCTGATGCAGGAAGTG<br>TTTACCGAATTTTTTCCGGGCTGCGTGGTGCATCCGTTTGGCAGCAGCATTAACAGCTTTGATGTGCATGGCT<br>GCGATCTGGATCTGTTTCTGGATCTGGGCGATCTGGAAGAACCGCAGCCGGTTCCGAAAGCACCGGAAAGC<br>CCGAGCCTGGATTCTGCACTGGCAAGCCCGCTGGATCCGCAAGCGCTGGCATGTACCCCGGCAAGTCCGCCG<br>GATAGCCAACCGCCGGCATCTCCGCAAGATTCTGAAGCACTGGATTTTGAAACCCCGAGCAGCAGCCTGGCA<br>CCGCAGACCCCGGATAGTGCACTGGCAAGTGAAACCCTGGCATCTCCGCAAAGCCTGCCGCCGGCAAGTCC<br>GCTGCTGGAAGATCGTGAAGAAGGCGATCTGGGCAAAGCGAGCGAACTGGCGGAAACCCCGAAAGAAGAA<br>AAAGCGGAAGGCGCGGCGATGCTGGAACTGGTGGGCAGCATTCTGCGTGGCTGTGTGCCGGGCGTGTATCG<br>TGTTCAGACCGTGCCGAGCGCGCGTCGCCCGGTTGTGAAATTTTGTCATCGCCCGAGCGGCCTGCATGGCGA<br>TGTGAGCCTGAGCAACCGCCTGGCGCTGCATAACAGCCGCTTTCTGAGCCTGTGCAGCGAACTGGATGGTCG<br>CGTTCGCCCGCTGGTTTATACCCTGCGTTGTTGGGCACAGGGTCGTGGTCTGAGCGGTAGCGGTCCGCTGCT<br>GAGTAATTATGCGCTGACCCTGCTGGTTATTTATTTTCTGCAGACCCGTGATCCGCCGGTTCTGCCGACCGTTA<br>GCCAGCTGACCCAGAAAGCGGGCGAAGGTGAACAGGTGGAAGTGGATGGCTGGGATTGCAGCTTCCCGCG<br>CGACGCCAGCCGCCTGGAACCGAGCATTAACGTGGAACCGCTGAGCAGCCTGCTGGCGCAGTTTTTTAGCT<br>GCGTGAGCTGCTGGGATCTGCGTGGCAGCCTGCTGAGCCTGCGTGAAGGTCAAGCGCTGCCGGTTGCGGGT<br>GGTCTGCCGAGCAATCTGTGGGAAGGTCTGCGTCTGGGTCCGCTGAATCTGCAGGATCCGTTTGATCTGAGC<br>CATAACGTGGCGGCGAATGTTACCAGCCGTGTTGCGGGTCGCCTGCAGAATTGCTGCCGTGCGGCGGCGAAT<br>TATTGTCGTAGCCTGCAGTATCAGCGTCGTAGCAGCCGTGGTCGTGATTGGGGTCTGCTGCCGCTGCTGCAAC<br>CGAGTAGCCCGAGTAGCCTGCTGTCTGCAACCCCGATTCCGCTGCCGCTGGCACCGTTTACCCAACTGACCG<br>CAGCGCTGGTTCAGGTGTTTCGCGAAGCGCTGGGCTGCCATATTGAACAGGCGACCAAACGTACCCGTAGC<br>GAAGGCGGTGGTACCGGCGAAAGCAGCCAGGGTGGCACCAGCAAACGCCTGAAAGTGGATGGCCAGAAAA<br>ACTGCTGCGAAGAAGGCAAAGAAGAACAGCAGGGCTGCGCGGGTGATGGCGGCGAAGATCGTGTGGAAGA<br>AATGGTGATTGAAGTGGGCGAAATGGTGCAGGATTGGGCGATGCAGAGCCCGGGTCAACCGGGTGATCTGC<br>CGCTGACCACCGGTAAACATGGCGCACCGGGTGAAGAAGGTCAGCCGAGTCATGCGGCGCTGGCAGAACGT<br>GGCCCGAAAGGTCATGAAGCAGCGCAGGAATGGAGCCAGGGTGAAGCGGGTAAAGGTGCAAGCCTGCCGA<br>GCTCTGCGAGCTGGCGTTGTGCGCTGTGGCATCGTGTTTGGCAGGGTCGTCGTCGTGCGCGTCGTCGTCTGC<br>AGCAGCAAACCAAAGAAGGCGCAGGTGGTGGTGCAGGTACCCGTGCAGGTTGGCTGGCAACCGAAGCACA<br>AGTTACCCAGGAACTGAAAGGCCTGAGCGGCGGTGAAGAACGTCCGGAAACCGAACCGCTGCTGAGCTTT<br>GTGGCGAGCGTGAGCCCGGCGGATCGTATGCTGACCGTGACCCCGCTGCAAGATCCGCAGGGTCTGTTTCCG<br>GATCTGCATCATTTTCTGCAGGTGTTTCTGCCGCAGGCGATTCGCCATCTGAAATAA
human TUT1 1-140 aa Q53A/R126A	ATGGCGGCGGTTGATAGCGATGTTGAAAGCCTGCCGCGCGGTGGCTTTCGCTGCTGCCTGTGCCATGTGACC<br>ACCGCGAATCGCCCGAGCCTGGATGCACATCTGGGTGGCCGCAAACATCGTCATCTGGTGGAACTGCGTGCG<br>GCGCGTAAAGCGGCGGGCCTGCGTAGCGTGTTTGTGAGCGGCTTTCCGCGCGATGTGGATAGCGCGCAGCTG<br>AGCGAATATTTTCTGGCGTTTGGCCCGGTGGCGAGCGTGGTGATGGATAAAGATAAAGGCGTGTTTGCGATT<br>GTGGAAATGGGCGATGTGGGCGCGCGTGAAGCGGTTCTGAGCCAGAGCCAGCATAGCCTGGGCGGTCATCG<br>TCTGCGTGTGCGTCCGGCGGAACAAAAAGAATTTCAGAGCCCGGCAAGCAAAAGCCCGAAA
human TUT1 1-140 aa F59A/F94A	ATGGCGGCGGTTGATAGCGATGTTGAAAGCCTGCCGCGCGGTGGCTTTCGCTGCTGCCTGTGCCATGTGACC<br>ACCGCGAATCGCCCGAGCCTGGATGCACATCTGGGTGGCCGCAAACATCGTCATCTGGTGGAACTGCGTGCG<br>GCGCGTAAAGCGCAGGGCCTGCGTAGCGTGGCGGTGAGCGGCTTTCCGCGCGATGTGGATAGCGCGCAGCT<br>GAGCGAATATTTTCTGGCGTTTGGCCCGGTGGCGAGCGTGGTGATGGATAAAGATAAAGGCGTGGCGGCGAT<br>TGTGGAAATGGGCGATGTGGGCGCGCGTGAAGCGGTTCTGAGCCAGAGCCAGCATAGCCTGGGCGGTCATC<br>GTCTGCGTGTGCGTCCGCGCGAACAAAAAGAATTTCAGAGCCCGGCAAGCAAAAGCCCGAAA
human TUT1 1-140 aa D90A/K91A	ATGGCGGCGGTTGATAGCGATGTTGAAAGCCTGCCGCGCGGTGGCTTTCGCTGCTGCCTGTGCCATGTGACC<br>ACCGCGAATCGCCCGAGCCTGGATGCACATCTGGGTGGCCGCAAACATCGTCATCTGGTGGAACTGCGTGCG<br>GCGCGTAAAGCGCAGGGCCTGCGTAGCGTGTTTGTGAGCGGCTTTCCGCGCGATGTGGATAGCGCGCAGCTG<br>AGCGAATATTTTCTGGCGTTTGGCCCGGTGGCGAGCGTGGTGATGGATAAAGCGGCGGGCGTGTTTGCGATT<br>GTGGAAATGGGCGATGTGGGCGCGCGTGAAGCGGTTCTGAGCCAGAGCCAGCATAGCCTGGGCGGTCATCG<br>TCTGCGTGTGCGTCCGCGCGAACAAAAAGAATTTCAGAGCCCGGCAAGCAAAAGCCCGAAA

Supplementary Table 3: Nucleotide sequences of RNAs

U6 snRNA	GUGCUCGCUUCGGCAGCACAUAUACUAAAAUUGGAACGAUACAGAGAAGAUUAGCAUGGCCCCUGCG CAAGGAUGACACGCAAAUUCGUGAAGCGUUCCAUAUUUU
U6_mini	GGAUACUAAAAUUGGAACGAUACAGAGUUCGCUCGUGAAGCGUUCCAUAUUUU
HDV ribozyme	GGGUCGGCAUGGCAUCUCCACCUCCUCGCGGUCCGACCUGGGCUACUUCGGUAGGCUAAGGGAGAAG

Supplementary Table 4: List of synthetic nucleotides

TUT1opt_D218A_Fw	GCGCTGTTTCTGGATCTGGGCGATCTGG
Tut1opt_D218A_Rv	CAGATCGCAGCCATGCACATCAAAGCTG
hTUT1opt_R38A_Rv	CGCGCCACCCAGATGTGCATCCAGGCTCG
hTUT1opt_K39A_Fw	GCGCATCGTCATCTGGTGGAACTGCGTGC
hTUT1opt_R50A_Rv	CGCCGCCGCACGCAGTTCCACCAGATGAC
hTUT1opt_Q53A_Fw	AAAGCGGCGGGCCTGCGTAGCGTGTTTGTG
hTUT1opt_Q53A_Rv	ACGCGCCGCACGCAGTTCCACCAGATGAC
hTUT1opt_D88A_Rv	CGCCATCACCACGCTCGCCACCGGGCCAAAC
hTUT1opt_K89A_Fw	GCGGATAAAGGCGTGTTTGCGATTGTGG
hTUT1opt_D90A_Fw	AAAGGCGTGTTTGCGATTGTGGAAATGG
hTUT1opt_D90A_Rv	CGCTTTATCCATCACCACGCTCGCCACC
hTUT1opt_K91A_Fw	GCGGGCGTGTTTGCGATTGTGGAAATGG
hTUT1opt_K91A_Rv	ATCTTTATCCATCACCACGCTCGCCACC
hTUT1opt_R126A_Fw	GAACAAAAAGAATTTCAGAGCCCGGCAAG
hTUT1opt_R126A_Rv	CGCCGGACGCACACGCAGACGATGACC
hTUT1opt_E127A_Fw	GCGCAAAAAGAATTTCAGAGCCCGGCAAG
hTUT1opt_E127A_Rv	GCGCGGACGCACACGCAGACGATGACC
hTUT1opt_R349A_Rv	GCCCGGCACACAGCCCGCCAGAATGCTGCCCACC
hTUT1opt_Q359A_Fw	GTGTATCGTGTTGCGACCGTGCCGAGCGCGCGTC
hTUT1opt_R357A_Rv	GACGCGCGCTCGGCACGGTCTGAACCGCATACACGCCCGGCAC
hTUT1opt_R374A_Fw	GCCCGGTTGTGAAATTTTGTCATGCGCCGAGCGGCCTGCATGG
hTUT1opt_R535A_Rv	CAGACCTTCCCACAGATTGCTCGGCAGACC
hTUT1opt_R535A_Fw	GCGCTGGGTCCGCTGAATCTGCAGGATCC
hTUT1opt_T556A_Rv	CGCAACATTCGCCGCCACGTTATGGCTCAG
hTUT1opt_R558A_Fw	AGCGCGGTTGCGGGTCGCCTGCAGAATTGC
Tut1opt_C19S_Fw	GTGGCTTTCGCTGCAGCCTGTGCCATGTGACC
Tut1opt_C19S_Rv	GGTCACATGGCACAGGCTGCAGCGAAAGCCAC
Slice_HDVr_pUC18vec_Fw	GGCTAGTCATAAGCTTGGCACTGGCCGTCGTTTTACAACG
Slice_U6FL4U_HDVr_Rv	GGAGATGCCATGCCGACCCAAAATATGGAACGCTTCACG
Slice_U6FL5U_HDVr_Rv	GGAGATGCCATGCCGACCCAAAAATATGGAACGCTTCACG
HDV_BamXhoHind_Fw_Slice	GGGTCGGCATGGCATCTCCACCTCCTCG
HDV_BamXhoHind_Rv_Slice	AAGCTTATGACTAGCCTCGAGAGCATGATCG
U6_190214_C3_Fw	TTCGCTCGTGAAGCGTTCCATATTTTTTGTCTTCC
U6_190214_C3_Rv	CTCTGTATCGTTCCAATTTTAGTATATGTG
pUC18seq_Ecoup	CGGCTCGTATGTTGTGTGGA
pUC18seq_Hindown	GCTGCAAGGCGATTAAGTTG
GCATG_T7pro_GGAUA_U6	GCATGTAATACGACTCACTATAGGATACTAAAATTGGAACGATAC
GCATGCATG_T7pro_GGAUAUA_U6	GCATGCATGTAATACGACTCACTATAGGATATACTAAAATTGG
GCATGCATG_T7pro_GGACTAA_U6	GCATGCATGTAATACGACTCACTATAGGACTAAAATTGGAACG
U6_222324CCC_Fw	TCCCCTAAAATTGGAACGATACAGAGAAG
U6_222324GGG_Fw	TGGGCTAAAATTGGAACGATACAGAGAAG
U6_toA20_Rv	TGTGCTGCCGAAGCGAGCACTATAGTGAGTCG
U6FL_A282930U_Fw	TTGGAACGATACAGAGAAGATTAGCATG
U6FL_delA27_Rv	TTTAGTATATGTGCTGCCGAAGCGAGC
U6FL_addA30a_Rv	TTTTTAGTATATGTGCTGCCGAAGCGAGC
U6FL_A28U_Rv	TTATAGTATATGTGCTGCCGAAGCGAGC
U6FL_A29U_Rv	TATTAGTATATGTGCTGCCGAAGCGAGC
U6FL_A30U_Rv	ATTTAGTATATGTGCTGCCGAAGCGAGC
HDVr_5ter_Fw	GGGTCGGCATGGCATCTCCACCTCCTCG
U6_snRNA_3ter3U_Rv	AAATATGGAACGCTTCACGAATTTGC
U6_snRNA_3terUAUU_Rv	AATATATGGAACGCTTCACGAATTTGC
snRNA_3terUUAU_Rv	ATAATATGGAACGCTTCACGAATTTGC
